# Asymmetrical accommodation in hyperopic anisometropic amblyopia

**DOI:** 10.1136/bjophthalmol-2017-310282

**Published:** 2017-10-19

**Authors:** Sonia Toor, Anna M Horwood, Patricia Riddell

**Affiliations:** 1 Academic Unit of Ophthalmology and Orthoptics, University of Sheffield, Sheffield, UK; 2 Infant Vision Laboratory, School of Psychology & Clinical Language Sciences, University of Reading, Reading, UK; 3 Orthoptic Department, Royal Berkshire Hospital, Reading, UK

**Keywords:** optics and Refraction, Vision, child health (paediatrics)

## Abstract

**Background/aims:**

To investigate the presence of asymmetrical accommodation in hyperopic anisometropic amblyopia.

**Methods:**

Accommodation in each eye and binocular vergence were measured simultaneously using a PlusoptiX SO4 photorefractor in 26 children aged 4–8 years with hyperopic anisometropic amblyopia and 13 controls (group age-matched) while they viewed a detailed target moving in depth.

**Results:**

Without spectacles, only 5 (19%) anisometropes demonstrated symmetrical accommodation (within the 95% CI of the mean gain of the sound eye of the anisometropic group), whereas 21 (81%) demonstrated asymmetrical accommodation. Of those, 15 (58%) showed aniso-accommodation and 6 (23%) demonstrated ‘anti-accommodation’ (greater accommodation for distance than for near). In those with anti-accommodation, the response gain in the sound eye was (0.93±0.20) while that of the amblyopic eye showed a negative accommodation gain of (−0.44±0.23). Anti-accommodation resolved with spectacles. Vergence gains were typical in those with symmetrical and asymmetrical accommodation.

**Conclusion:**

The majority of hyperopic anisometropic amblyopes demonstrated non-consensual asymmetrical accommodation. Approximately one in four demonstrated anti-accommodation.

## Introduction

Evidence suggests that accommodation is symmetrical in each eye,^1–5^ so in anisometropia, the least ametropic eye determines the amount of accommodation, with the amblyopic eye ‘lagging behind’.^6–10^ However, some report that subtle asymmetrical accommodation can occur in typical, young adults,^11 12^ demonstrating that there is a mechanism to drive different responses in each eye separately.

Asymmetrical accommodation, however, has rarely been considered clinically. Although reduced accommodation has been reported in amblyopic eyes (overview in von Noorden and Campos p. 260^13^), accommodation in clinical and research settings is generally tested monocularly, and so asymmetrical accommodation would have gone undetected. Reduced accommodation in the amblyopic eye could be ascribed to reduced visual acuity (VA) or sensory loss over the central retinal region^7 8^ due to monocular contrast deprivation in anisometropia.^14^

A case study from our lab, where vergence and binocular accommodation were assessed simultaneously and continuously, reported a child with hyperopic anisometropic amblyopia, who demonstrated an extreme example of asymmetrical accommodation.^15^ Without their spectacle correction, the sound eye accommodated appropriately for target distance but the amblyopic eye repeatedly ‘anti-accommodated’ (accommodation in the wrong direction for the change in target distance), showing a greater accommodation response in the distance than at near.

The aim of this study was to determine whether the presence of asymmetrical accommodation was more widespread in hyperopic anisometropic amblyopia.

## Methods

The prospective study adhered to the Declaration of Helsinki and obtained both University and National Health Service ethics approval. Informed consent was obtained from parents and age-appropriate assent from the children.

### Participants

Hyperopic anisometropic amblyopes, aged 4–8 years, were recruited from an Orthoptic department. All had been assessed with cycloplegic retinoscopy, fundus and media check and an orthoptic examination. All had been prescribed fully corrected spectacles, worn full time for at least 6 weeks. Amblyopia was defined by the VA in either eye. All had VA in the amblyopic eye worse than 0.2 logarithm of the minimum angle of resolution (logMAR), VA in the non-amblyopic eye of at least 0.2 logMAR, with >0.1 logMAR interocular difference.

A control group with a mean age matched to the patient group was recruited from the University of Reading typically developing Child Development Group database. All had VA ≥0.2 logMAR in each eye, with no more than 0.1 logMAR interocular difference.

### Laboratory testing

**Figure 1:**
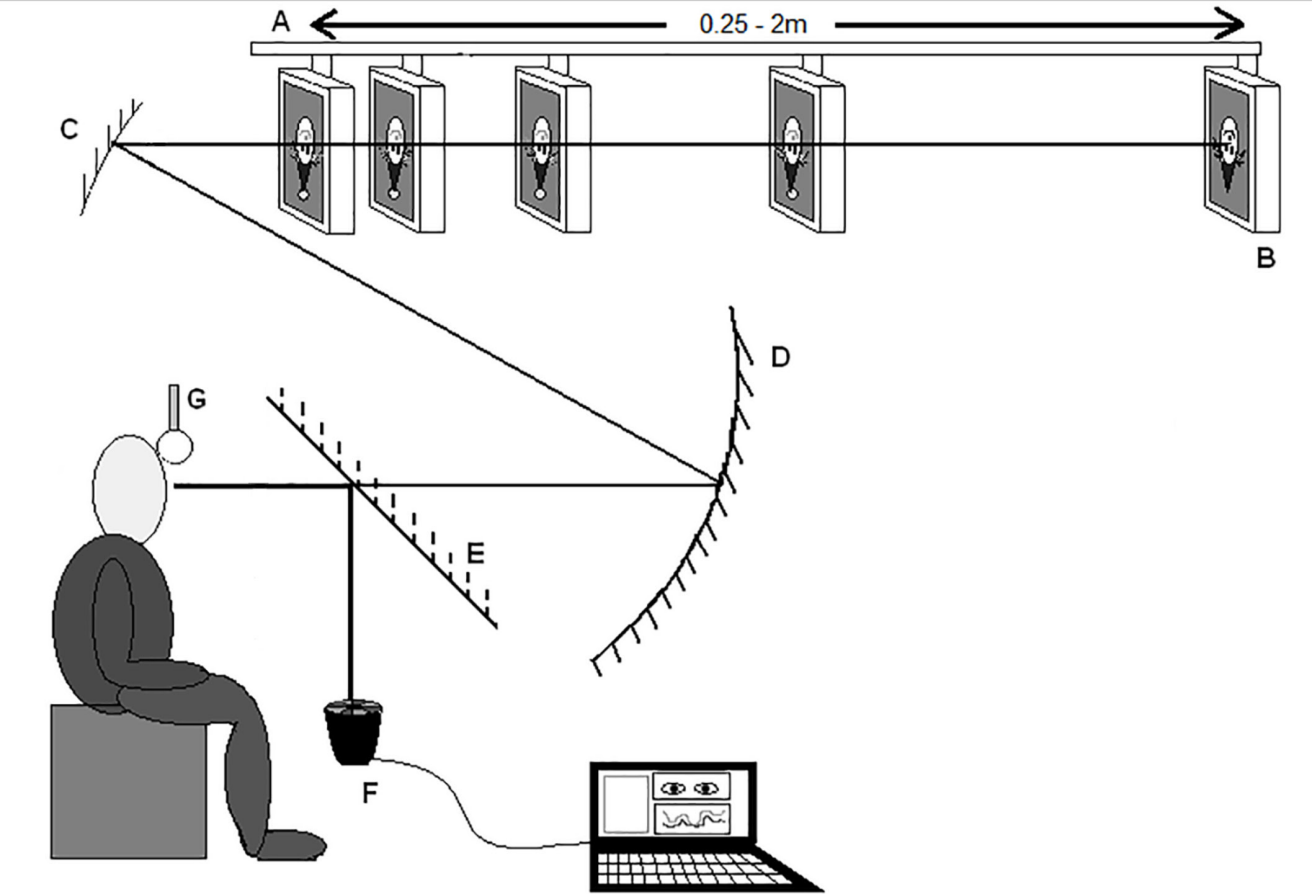
Infant vision laboratory. (A) Motorised beam. (B) Target monitor. (C) Upper concave mirror. (D) Lower concave mirror. (E) Hot mirror. (F) PlusoptiX SO4 PowerRef II. (G) Headrest.

A PlusoptiX SO4 photorefractor in PowerRef II mode made simultaneous and continuous refraction and eye position recordings in both eyes at 25 Hz. The target was a detailed cartoon picture of a clown’s face subtending 3.15° at 2 m, which contained detailed elements down to 1 screen pixel but were easily identifiable even with reduced VA. Instructions were minimal, children were simply asked to ‘watch the clown’. The target was presented via a mirror arrangement (figure 1). Measurements were taken at five fixation distances in a pseudo-random order (0.33, 2, 0.25, 1 and 0.5 m), representing demands of 3 dioptres (D), 0.5 D, 4 D, 1 D and 2 D, respectively. Data at 4 D were discarded due to reasons such as unacceptable data loss from pupil miosis. The procedure is explained in detail in the online supplementary file 1 and previous papers.^16 17^

10.1136/bjophthalmol-2017-310282.supp1 Supplementary file 1

The testing procedure was repeated twice in the same session, both with and without spectacles, and results averaged for each condition. Data without spectacles were collected first to ensure maximal cooperation as this was considered to be more valuable in the context of the study.

Vignettes of data representing one continuous second of stable data (25 data points) at each fixation distance were chosen for analysis (further details can be found in a previous paper^16^). A bespoke Excel macro converted refraction into accommodation (in D), and eye position into convergence (in metre angles (MA)), with appropriate corrections for lab calibration studies and individual interpupillary distance and angle lambda.^16^ If spectacles were worn, an additional correction was applied to appropriately correct for spectacle magnification. See online supplementary file 1 for further details about data processing and calibration.

From measurements at the four fixation distances, the gain of the accommodation response in relation to target demand was calculated for each eye (where a gain of 1.0 infers a perfect response to the stimulus). The 95% confidence intervals (CIs) of the mean gain of the sound eye for the anisometropic group were used to provide the range of typical responses. If accommodation was symmetrical, the gain would be similar in each eye. If accommodation gain in the amblyopic eye fell outside these CI, accommodation was asymmetrical.

Data were analysed with SPSS V.22 software using one-way repeated-measures analysis of variance (ANOVA), two-way mixed factor ANOVA (distance as the within-subject factor and group as the between-subject factor) with post-hoc t-tests where required (Bonferroni corrected). If assumptions of sphericity were violated, the Greenhouse–Geisser statistics were quoted.

## Results

### Participants

All 30 participants with hyperopic anisometropic amblyopia were Caucasian. Four anisometropes were excluded. One anisometrope was myopic in one eye and therefore might have different accommodative demands, one was wearing the incorrect prescription and two failed to accommodate in either eye, so it was unclear whether they were attending to the task. The details of the remaining 26 anisometropes are summarised in table 1 and will be discussed further, along with the response to occlusion therapy, in a separate paper.

**Table 1:** Summary of details of the hyperopic anisometropic amblyopes

	Mean±95% CI	Range
Age (years)	5.65±0.39	4 to 8
VA amblyopic eye (LogMAR)	0.69±0.13	0.275 to 1.75
VA amblyopic eye (Snellen equivalent)	20/98	20/38 to 20/1125
DS sound eye (D)	+1.66 ±0.33	+0.25 to +3.75
DC sound eye (D)	+0.08 ±0.10	−0.50 to +1.00
MSE sound eye (D)	+1.69 ±0.34	+0.25 to +3.75
DS amblyopic eye (D)	+4.57 ±0.38	+3.00 to +6.50
DC amblyopic eye (D)	+0.36 ±0.30	−1.25 to+2.00
MSE amblyopic eye (D)	+4.72 ±0.33	+3.25 to+6.25
Anisometropia (D)	3.03±0.40	1.75 to 5.75

D, dioptres; DC, dioptres cylinder; DS, dioptres sphere; logMAR, logarithm of the minimum angle of resolution; MSE, mean spherical equivalent; VA, visual acuity.

Fifteen controls were recruited but two were excluded (6.08±0.35 years; range 5–7 years). One participant had long eyelashes, prohibiting data recording and one had previously undetected hyperopia. These children were not refracted under cycloplegia but all had no more than 1.5 D of hyperopia (measured using the PlusoptiX) and VA within normal limits (right eye (RE): 0.04±0.04 logMAR, range −0.075 to 0.15 logMAR; left eye (LE): 0.03±0.04 logMAR, range −0.05 to 0.125 logMAR).

### Types of accommodation response without spectacles

**Figure 2:**
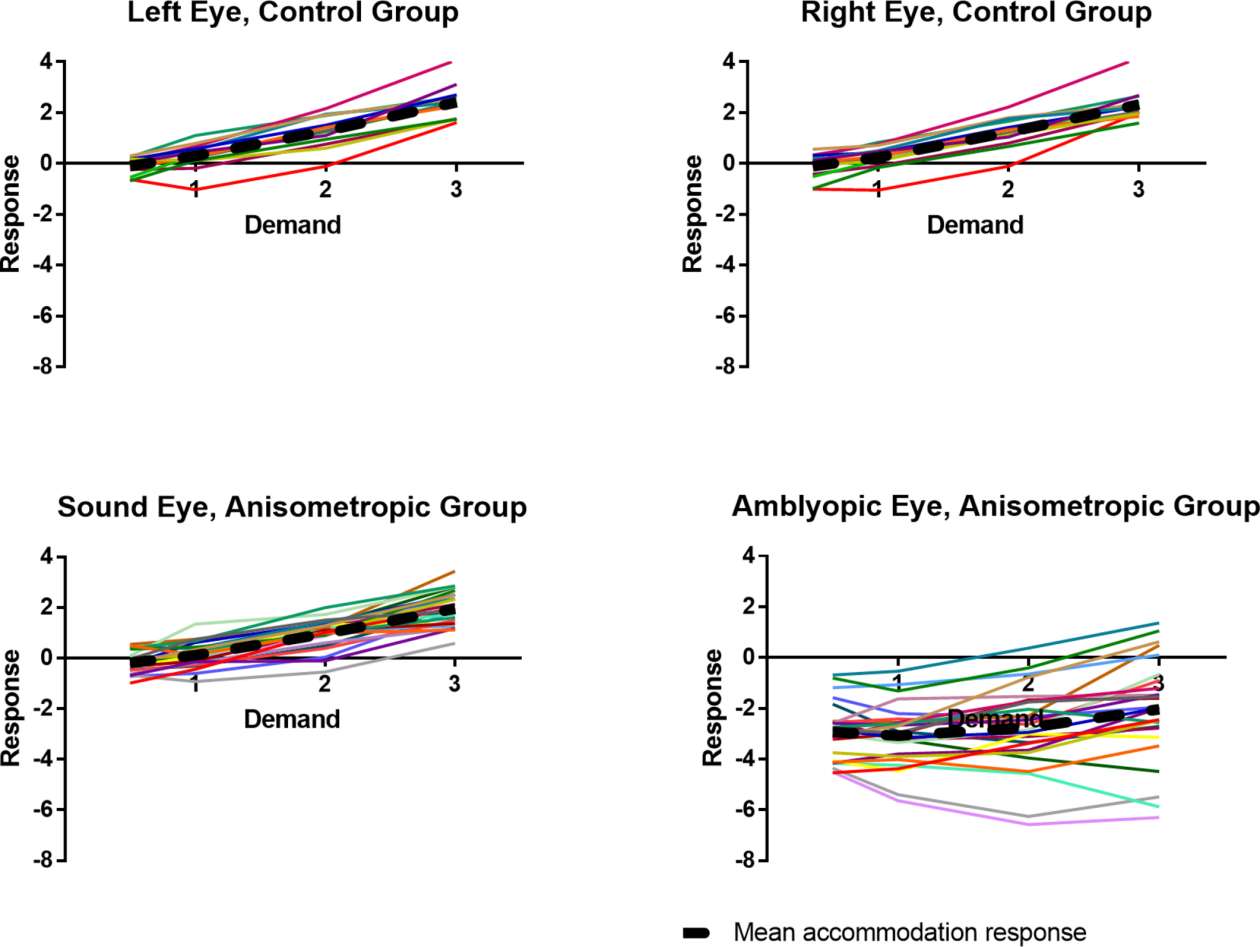
The accommodation response in the sound/left eye and amblyopic/right eye for each individual in the control and anisometropic group. The black lines denote the mean response.

Figure 2 illustrates the individual accommodative gains of each eye in the anisometropic and control groups. In the control group, there was no significant difference in mean accommodation gain between the eyes (RE: 0.98±0.12; LE: 1.01±0.12; t(12) = −0.57, p=0.576). Across the anisometropic group, the mean accommodation gain in the sound eye (0.86±0.08) and the amblyopic eye (0.41±0.22) was significantly different (t(25) = 4.12, p=0.00).

**Figure 3:**
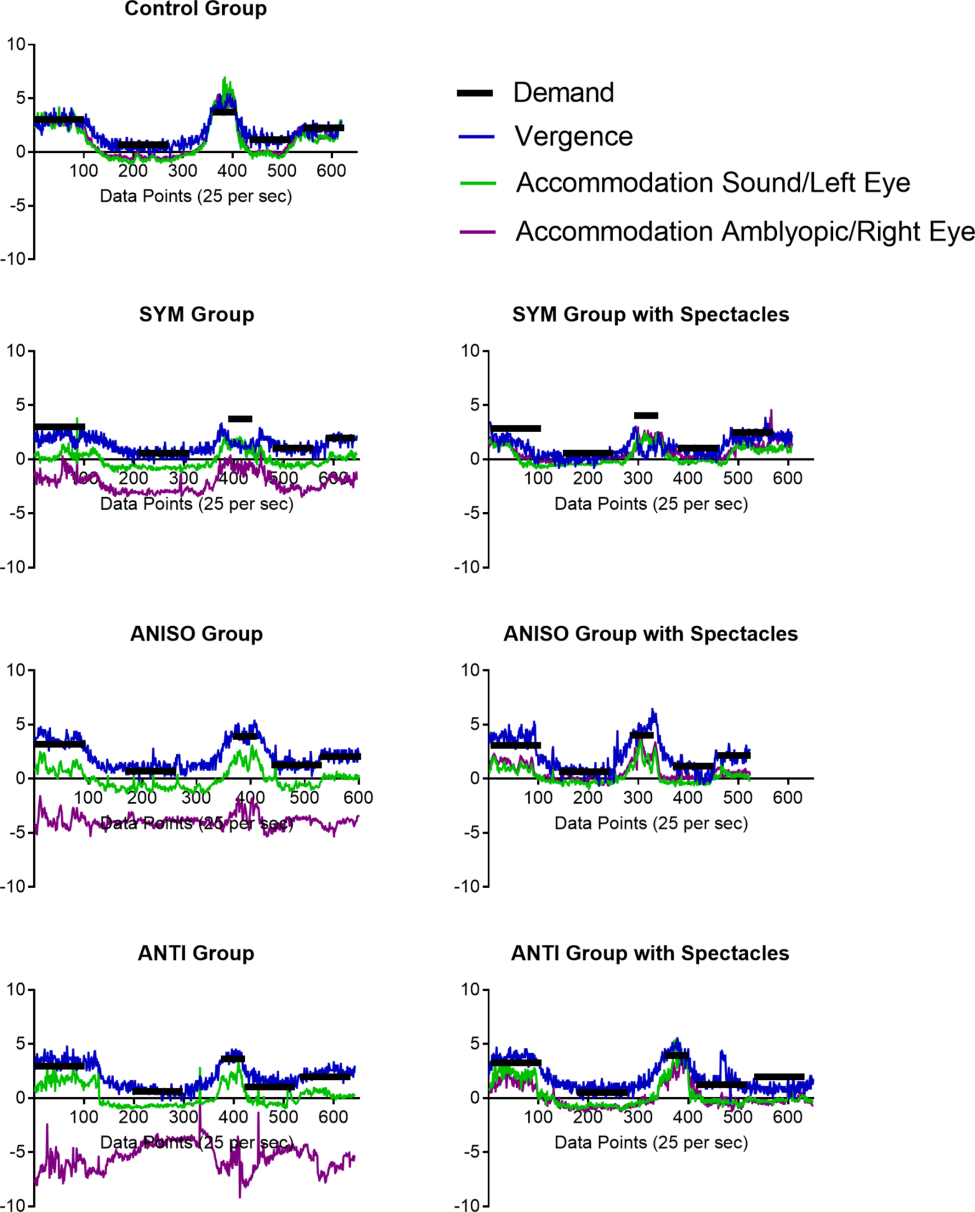
An example of a participant in each of the four groups (control, SYM, ANISO and ANTI) to demonstrate the types of accommodation response with and without spectacles. The black lines represent demand of the target moving from a demand of 3 D, to 0.5 D, 4 D, 1 D and then 2 D. The blue lines represent convergence. The green line represents the accommodation response in the sound eye of the anisometropes (left eye of control participant). The purple line represents the accommodation response in the amblyopic eye of the anisometropes (right eye of control participant). The separation between the green and purple line denotes anisometropia. In the SYM group, the difference in accommodation response between the sound eye and amblyopic eye was similar across target distances. The ANISO group had a larger accommodative difference between the eyes at near than in the distance as the amblyopic eye accommodated less. In the ANTI group, the sound eye accommodated appropriately but the amblyopic eye *relaxed* for near fixation. ANISO, aniso-accommodation; ANTI, anti-accommodation; SYM, symmetrical accommodation.

The 95% CI for accommodation gain in the sound eye of the entire anisometropic group was ±0.08. Individual values for difference in gain between the eyes were then compared with this value, which equates to no more than 0.25 D difference in accommodation between the eyes at 0.33 m. Accommodation gain in the amblyopic eye within the 95% CI of the mean gain of the sound eye (<±0.08) was defined as symmetrical, and if outside the 95% CI (>±0.08), was defined as asymmetrical. Five anisometropes (19%) fulfilled the criterion of symmetrical accommodation (SYM group). Twenty-one (81%) anisometropes had a difference in mean accommodation gain >0.08 and therefore had asymmetrical accommodation. Fifteen (58%) of these had a positive accommodation gain in both eyes but with greater gain in the sound eye, that is, the amblyopic eye underaccommodated for near, and were categorised as having aniso-accommodation (ANISO group). Six anisometropes (23%) had a negative accommodation gain in the amblyopic eye (accommodation greater for distance than near) and were categorised as having anti-accommodation (ANTI group). This method of defining the type of accommodation response was used to investigate the control group. Five controls (38%) demonstrated symmetrical accommodation and eight demonstrated aniso-accommodation (62%). No controls had anti-accommodation. Those controls with aniso-accommodation had a mean difference in gain between the eyes of 0.04 (±0.15), which was significantly lower than the difference of 0.23 (±0.14) found in the ANISO group (t(21) = −2.374, p=0.027). An example patient from each of the three anisometropic groups (with and without spectacles) and the control group is displayed in figure 3.

All groups had close to the ideal gain of 1.0 in the sound eye (controls (LE): 1.01±0.12; SYM: 0.85±0.18; ANISO: 0.85±0.18; ANTI: 0.93±0.20). Similarly, the accommodation gain was close to 1.0 in the fellow eye in the control group (RE: 0.98±0.12) and amblyopic eye in the SYM group (0.82±0.21). In comparison, the ANISO group had a reduced accommodative gain in the amblyopic eye (0.55±0.21). The ANTI group showed a negative accommodative gain in the amblyopic eye (−0.44±0.23).

An ANOVA revealed no significant difference in the accommodative gain between the sound eye of the three anisometropic groups and the control group (LE) (F(3,35) = 1.42, p=0.253). For the amblyopic eye (RE in the control group), there was a significant main effect between the four groups (F(3,35) = 27.41, p<0.001). The accommodative gain in the amblyopic eye of the ANTI group was significantly different to each of the other groups (all p<0.001).

**Figure 4:**
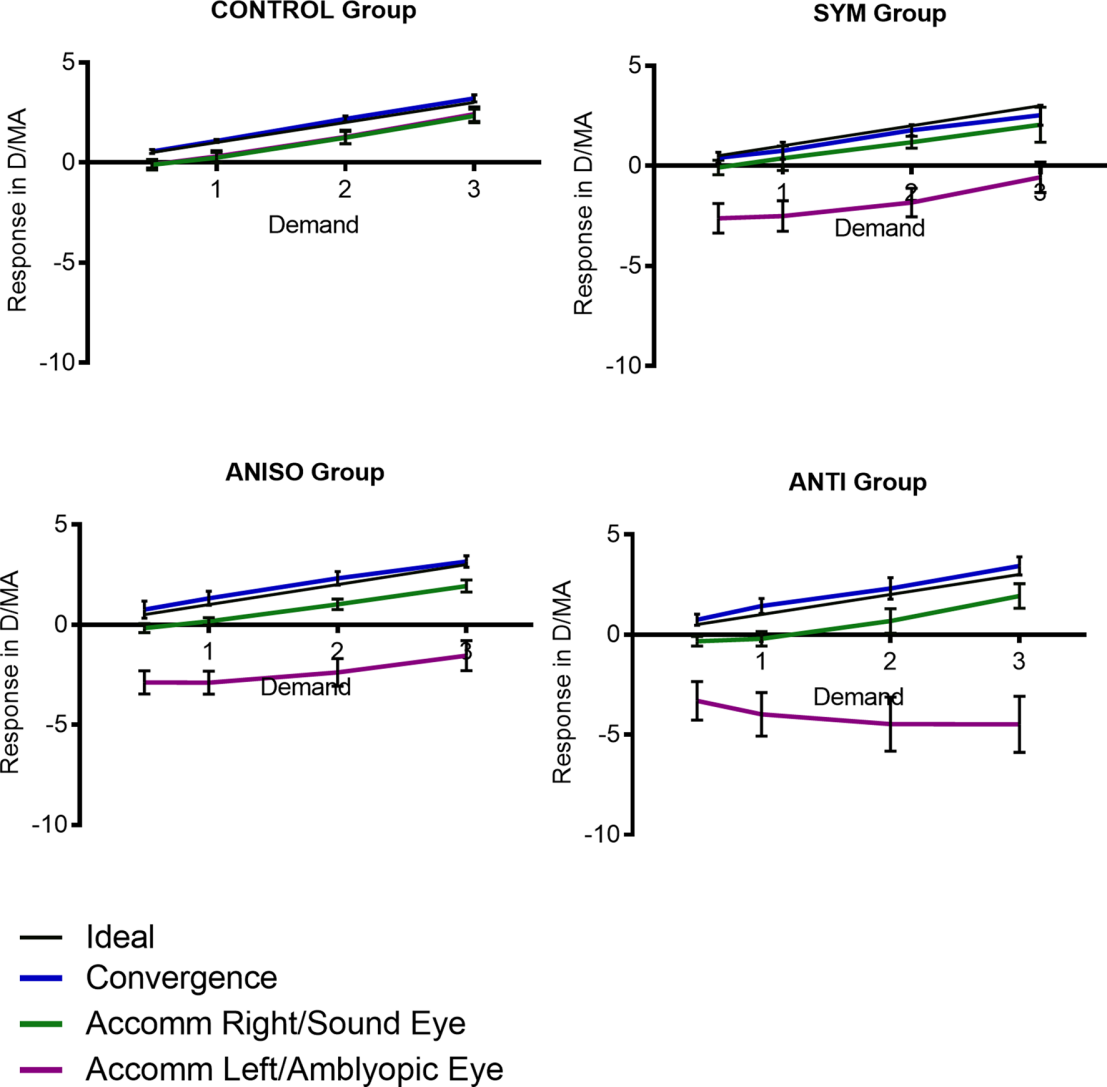
Vergence and accommodation responses in the control group and each anisometropic group without glasses for demands of 0.5 D, 1 D, 2 D and 3 D. In all the graphs, the grey line represents an ideal response and the vergence response is in blue. In the control group, the green line and purple line represent the accommodation response of the right eye and the left eye, respectively. In the anisometropic groups, the green and purple lines represent the accommodation response in the sound eye and amblyopic eye respectively. Error bars denote ±95% CI. ANISO, aniso-accommodation; ANTI, anti-accommodation; SYM, symmetrical accommodation.

Mean accommodation responses in each eye for the control and anisometropic groups are illustrated in figure 4. In the SYM group, the difference in accommodation response between the sound eye and amblyopic eye was similar across target distances (2.54 D at 2 m and 2.63 D at 0.33 m; which is similar to their mean cycloplegic anisometropia of 2.85 D). At 2 m, the ANISO group had a similar accommodative difference between the eyes of 2.71 D (with mean cycloplegic anisometropia of 2.53 D). However, the difference increased at 0.33 m to 3.47 D as the amblyopic eye accommodated less. The ANTI group also had a similar accommodative difference at 2 m of 2.98 D (despite a greater mean cycloplegic anisometropia of 4.42 D) but at 0.33 m the difference more than doubled to 6.41 D as the amblyopic eye *relaxed* for near fixation.

The difference in accommodative response between the eyes at 0.5 D was compared with the difference at 3 D for the three anisometropic groups and the control group. There was an overall significant difference between the groups (F(1,3) = 256.71, p<0.001), a significant main effect of distance (F(1,35) = 62.99, p<0.001) with a greater difference at 3 D than at 0.5 D and a significant distance*group interaction (F(3,35) = 28.48, p<0.001). The difference in accommodation response at 33 cm in the control group was significantly different from each of the anisometropic groups (all p<0.001) and the ANTI group responses were significantly different from the other anisometropic groups (vs SYM: p=0.003; vs ANISO: p=0.005).

### Accommodation response with spectacles

With spectacles, the 95% CI of the sound eye of the entire anisometropic group was 0.09. On comparison of the amblyopic eye to this value, 8 anisometropes (32%) had symmetrical accommodation, 17 (68%) had aniso-accommodation and no anisometropes demonstrated anti-accommodation (no data were collected from one child) but still demonstrated some aniso-accommodation. Figure 3 illustrates the effect of wearing spectacles for each patient example in each group.

### Vergence

Vergence gains were typical^16 18^ (control: 1.07±0.07; SYM: 0.88±0.24: ANISO 0.97±0.11; ANTI: 1.04±0.10) with no significant difference between the four groups (F(3,35) = 1.71, p=0.183). Therefore, both eyes were fixating the target and any difference in refraction cannot be ascribed to off-axis errors.

## Discussion

The majority of the hyperopic anisometropic amblyopes had asymmetrical accommodation. Fifty-eight per cent had aniso-accommodation, with greater accommodative lag in the amblyopic eye at near. More interestingly, 23% of anisometropes demonstrated *anti-*accommodation. The sound eye accommodated appropriately when viewing a near target but the amblyopic eye accommodated in the opposite direction with a greater accommodation response at distance than at near. This finding indicates that the child with anti-accommodation reported by Horwood and Riddell^15^ is not a unique case. Only 19% of anisometropes were found to have symmetrical accommodation, contradicting previous literature.^6–10^

It is very possible that other researchers and clinicians have overlooked the existence of asymmetrical accommodation, as objective accommodation is usually measured monocularly, even under binocular conditions. There is an assumption that testing one eye reflects the response of both eyes. Any reduced accommodation response in the amblyopic eye, as discussed by von Noorden and Campos,^13^ could be ascribed to reduced VA in a fixing amblyopic eye, driving weak accommodation in both eyes.

Some studies have tried to induce aniso-accommodation but results have been negative, weak or fleeting.^1–3 11 12^ The naturally occurring, long-term abnormal input of developmental anisometropia is a more extreme visual experience than is possible to induce experimentally and may enable such responses to develop.

Although the ANISO group continued to underaccommodate somewhat for near, it was dramatic that the anti-accommodation resolved with spectacle correction. This suggests that anti-accommodation is not hardwired, but more driven by visual input and subject to short-term variation.

Our data only allow us to speculate on possible mechanisms. It is difficult to account for anti-accommodation with the current models in which both eyes are driven by a single accommodative signal. It becomes easier to explain this condition if accommodation is driven independently. The anti-accommodation might be explained by a misinterpretation of blur cues in the amblyopic eye. An alternate explanation is that the anti-accommodation is the result of an active strategy that avoids conflict between a clear image in the sound eye and a less clear image in the amblyopic eye. For distant targets, where accommodative demand is low for both eyes, some accommodative effort could be made in the amblyopic eye to compensate for the anisometropia. On viewing a near target, however, while the blur signal to the sound eye would result in appropriate accommodation to clear the image, the necessary accommodative effort required to both accommodate for near and overcome the hyperopia might be too great for the amblyopic eye to compensate. Rather than partially accommodating, this might result in total relaxation of the amblyopic eye therefore producing anti-accommodation. Full correction of the anisometropic blur with spectacles would reduce the accommodative effort required by the amblyopic eye and hence make it possible for the amblyopic eye to accommodate.

The ability to make simultaneous measurements of accommodation in each eye and confirm on-axis refraction by measuring simultaneous vergence allowed us to find behaviour, which may have been missed by other methods. The study has some limitations but they are unlikely to significantly affect the results. We were unable to make individual calibrations of refraction in these children. There is evidence that group means are acceptable for studies such as these,^19^ but individual responses and gains may be more variable than the mean data suggest. The limited linear operating range of the photorefractor may have caused further inaccuracies in refraction measurements as calculations become non-linear towards these limits. However, our calibration studies on older children and adults and those published by others^19^ suggest that the PlusoptiX photorefractor is more likely to underestimate refraction, than overestimate it at these limits. This suggests that anti-accommodation would be even more marked than reported in this paper.

The majority of the control group also demonstrated aniso-accommodation to some extent. This is unlikely to be due to calibration error as the calibration factor should not differ in either eye. The results suggest that subtle aniso-accommodation in normals might be more common than previously thought.^1–3 11 12^ We did not refract the controls under cycloplegia, but during lab testing we determine the maximum hyperopic refraction found at any time in the session, which correlates extremely well with cycloplegic refraction.

This is a small-scale study with the possible consequence that some of the statistical analysis may have been underpowered. The finding of any significant differences even in this relatively small group suggests that anti-accommodation in hyperopic anisometropic amblyopia is genuine and worthy of further study. Our findings provide clear evidence that accommodation in not necessarily a consensual response and provides further support that children should be wearing their full cycloplegic prescription to avoid aniso-accommodation and anti-accommodation.

## Conclusion

The majority of children with hyperopic anisometropic amblyopia have asymmetrical, rather than symmetrical, accommodation without spectacles, refuting previous suggestions in the literature.^1–5 10^ The majority of these children have aniso-accommodation but 23% anti-accommodate. This suggests that there must be a mechanism by which it is possible to drive accommodation in each eye independently, even if this is rarely necessary in the general population.
