# Supplementary material for: Asymmetrical accommodation in hyperopic anisometropic amblyopia
Source: Br J Ophthalmol. 2017 Oct 19;102(6):772–8. doi: 10.1136/bjophthalmol-2017-310282 (PMC5787021; doi:10.1136/bjophthalmol-2017-310282)
Supplement: Supplementary file 1 [file bjophthalmol-2017-310282supp001.pdf]

Asymmetrical accommodation in hyperopic anisometropic amblyopia

Horwood, Toor, Riddell
Supplemental Materials

Details and Validation of Method

Apparatus

The remote haploscopic videorefractor (RHV) allows for the objective assessment of accommodation and convergence whilst targets are presented at five fixation distances ranging from 0.25 to 2m. The RHV consists of two optical pathways: one for off-axis infra-red (IR) continuous photorefraction at a speed of 25Hz and one for target presentation.

The photorefraction pathway incorporates a PlusoptixSO4 PowerRefII photorefractor (Plusoptix GmbH,Nurnberg, Germany) which makes simultaneous measurements of eye position and refraction, which are converted into vergence and accommodation measurements. Data is collected via a 600mm diameter 'hot' mirror, set at 45°, which reflects IR light but is transparent to visible light, thus the camera sensors can be placed in the same optical plane as the target but without obscuring it.

The targets are viewed through two concave mirrors (Figure 1 C and D) so that targets are presented on the beam above the participant (Figure 1 A) but are viewed as a virtual image at eye level. Participants see the virtual target move back and forth at eye level and are unable to see the true position of the target as the equipment is enclosed in black shuttering except for the opening through which the virtual target is visible.

Targets are presented on a high resolution computer monitor (Figure 1 B) mounted on the motorised beam (Figure 1 A) which moves at a speed of 0.4 metres per second and stops at 5 different fixation distances of 0.25m, 0.33m, 0.5m, 1m and 2m. These distances correspond to demands of 4, 3, 2,1 and 0.5 dioptres/meter angles (D/MA) respectively. The targets are presented in a pseudo-random order so that near and distant presentations are alternated (3 followed by 0.5, 4, 1, and then 2D/MA). Vergence is measured in MA to enable direct comparisons between accommodation and vergence responses in relation to target demand more accurately between participants with different inter-pupillary distances. During movement of the target the motor (placed at 2.75m from the participant) can be heard, which therefore alerts the participant of the target movement, but gave no clues to the direction of movement or the target position.

Testing is done in low light levels but despite this there is often an unacceptable level of data loss at 0.25m/4D due to pupil constriction. At this distance there is also the possibility of inaccurate measurements due to more off-axis differences in peripheral refraction as the eyes converge. The vertical offset of the concave mirrors also induces cylindrical errors, which at 3D is less than 0.5DC, so does not significantly affect acuity and considered acceptable, but this exceeds 0.5DC at 4D. In addition, the lower concave mirror is seen in physiological diplopia, with the screen visible within the

overlap of the two diplopic images. At 4D this physiological diplopia overlap is smaller and just excludes the very far edges of the image of the whole screen. This presents a slightly different fusional stimulus as the extreme target screen edges are not fully visible within the physiological diplopic images of the lower image (although the target itself is still well within the overlap). Finally, we also find that many participants simply do not converge or accommodate to the 4D stimulus, despite typical clinical convergence responses. Due to these reasons the data at 0.25m/4D are discarded but the target distance was retained in the testing sequence so that a distance target is always presented after a near one and vice versa, which makes interpretation of the data output easier and clearer.

Target

The target was a detailed picture of a clown's face (Supplemental material figure 1), to stimulate accommodation as in a real-life situation. It was originally designed to maintain attention in young children with a range of visual acuities. The target subtended a visual angle of 3.15° at 2m and 18.26° at 33cm. It is a high contrast target and contains a wide range of spatial frequencies. The clown's eyes, mouth and hat provide the lower spatial frequencies, whilst the nose (3mm wide, subtending 5 minutes of arc at 2m and 30 minutes of arc at 2m) and outlines (1 pixel wide, subtending 1 minute of arc at 2m) provide the higher spatial frequencies. The nose, eyes and mouth change to produce a second version of the clown which alternate at 1Hz.

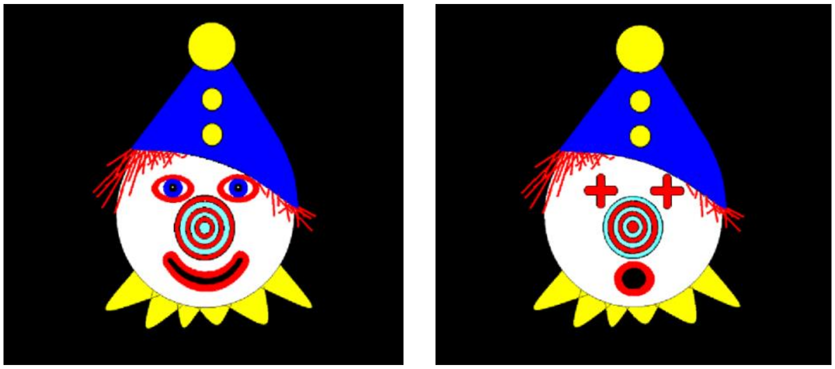

Figure 1 Clown target.

The whole apparatus is enclosed in a matte black shuttering therefore excluding peripheral stimuli. However, the background luminance of the screen (10cd/m^2) is slightly visible against the screen edges, which could possibly create a stimulus to accommodation. To minimise this, the screen edges are masked by using an increasing density acetate filter to blur the edge contrast gradient.

Data Collection

Testing took approximately 25 minutes for the entire session. Of this time, approximately 10 minutes was spent on data recording. If the participant was co-operating, the data with spectacles was taken immediately after the data without spectacles. No more than 5 minutes break was given between data collection.

The participant's eyes are observed during recording to ensure at least 2 seconds of continuous steady fixation before moving onto the next distance. Data recording can also be interrupted by excessive blinking, long eyelashes, droopy eye lids, spectacle reflections, small pupils and large pupils. In these instances, the duration of recording was increased until 2 seconds of steady data is achieved.

No specific instructions are given to the participants except to simply look at the target. It is important to assess naturalistic responses as Horwood and Toor [1] and Horwood et al [2] highlight that accommodation measures are prone to large instruction and effort effects.

Data Processing

A macro was designed to process the raw data and calculate accommodation and vergence responses over time. Refractive error is converted to accommodation by conversion of negative to positive data and vice versa. For example, -3DS myopic refraction becomes 3DS of accommodation. Eye position, which consists of an adduction measure in one eye and abduction in the other eye, is converted to vergence by conversion on negative and positive values of only the abduction measurements. The addition of both eyes gives the total vergence. The vergence data is converted from degrees into MA using individual interpupillary distance (IPD). This allows vergence and accommodation to be directly comparable on the same scale in relation to target demand.

The macro detects and removes accommodation and vergence data before and after spikes which appear due to blinks. It also removes patches of missing data.

The accommodation and vergence data is displayed in a chart format to visually identify vignettes of 25 data points (1 second of continuous stable data) at each fixation distance. These are averaged and the macro corrects for calculated individual angle kappa and IPD which is required to calculate vergence in MA. As we are unable to test at infinity both, angle kappa and IPD, are calculated by best estimate with the data from 4 fixation distance extrapolated to the slope intercept of the y-axis [3]. Calculation of accommodation depends on brightness gradient, and therefore is unaffected by spectacle correction. Vergence is calculated by horizontal image shift in screen pixels and therefore is affected by any magnification produced by spectacles. In these cases, a magnification correction was applied to either eye, which corrected for possible errors in estimates of right and left eye position depending on the strength of the anisometropic spectacles.

Inter- scorer reliability on masked scoring where each scorer was free to choose the vignette was excellent. For both vergence and accommodation, this analysis showed a high agreement: for vergence: r=0.99, mean inter-scorer difference (±95%CI) = 0.037 (±0.37) MA; for accommodation r=0.99, mean inter-scorer difference (±95%CI) = 0.0095 (0.175) D.

Calibration

Individual refraction calibration has been recommended by some authors [4] as they state that the calculated refraction of one person in response to a known amount of defocus will differ slightly (and may also be subject to ethnic differences [5]). Calibration depends on comparing the Plusoptix reported refraction in an eye that cannot see the target (in their case using a visible light filter) through a known lens, and comparing it with the fixating eye without a lens. We did not carry out such calibration for multiple reasons. Firstly, it was rarely possible in children due to the long testing session. In the situation here, where we are reporting comparisons between the eyes of the same children, unless each eye had a different calibration factor, calibration errors are unlikely to account for our findings because we are looking for a *change* in refraction between eyes at different target distances. The most extreme calibration difference could not account for the anti-accommodation we found.

When setting up the lab, we ascertained a group calibration factor for the lab by comparing the Plusoptix with dynamic retinoscopy at different distances, which is used for all initial estimates of refraction. We did carry out further calibration studies on a different group of children and young adults because Bharadwaj et al [4] reported non-linearity of refraction towards the edges of the apparatus operating ranges Research within the lab shows the PlusoptiX produces a linear response to refractive errors with refractive errors between +5.5D and -4D with very small inter-participant differences. This is equivalent to 5.5D of myopia and 4D of hypermetropia and therefore similar to the reported operating range of the PlusoptiX [6-7]. Beyond this, the photorefractor provides an underestimation, rather than an over-estimation, of refraction. This is a further reason why calibration errors could not explain our findings. This further study confirmed that after the group correction, the slopes of our lab responses were not significantly different from the manufacturer’s readings, and Blade & Candy [8], using similar apparatus have shown that group means in infants and adults are similar.

References

1. Horwood AM, Toor SS. Clinical test responses to different orthoptic exercise regimes in typical young adults. *Ophthalmic and Physiological Optics* 2014;34:250-262.

2. Horwood AM, Toor SS, Riddell PM. Change in convergence and accommodation after two weeks of eye exercises in typical young adults. *J AAPOS* 2014;18(2):162-168.
3. Horwood AM, Riddell PM. The use of cues to convergence and accommodation in naive, uninstructed participants. *Vision Res* 2008;48(15):1613-24.
4. Bharadwaj SR, Sravani NG, Little J, et al. Empirical variability in the calibration of slope-based eccentric photorefraction. *J Opt Soc Am A Opt Image Sci Vis* 2013;30(5):923-931
5. Sravani NG, Nilagiri VK, Bharadwaj SR. Photorefraction estimates of refractive power varies with the ethnic origin of human eyes. *Sci Rep* 2015;5:7576
6. Choi M, Weiss S, Schaeffel F, et al. Laboratory, clinical, and kindergarten test of a new eccentric infrared photorefractor (PowerRefractor). *Optom Vis Sci* 2000;77(10): 537-548.
7. Gabriel GM, Mutti DO. Evaluation of infant accommodation using retinoscopy and photoretinoscopy. *Optom Vis Sci 2009;86*(3):208-215.
8. Blade PJ, Candy TR. Validation of the PowerRefractor for measuring human infant refraction. *Optom Vis Sci 2006;83*(6):346-353.
